# Crystal structure of 13-(*E*)-(2-amino­benzyl­idene)parthenolide

**DOI:** 10.1107/S2056989018013622

**Published:** 2018-10-09

**Authors:** Shobanbabu Bommagani, Narsihma R. Penthala, Sean Parkin, Peter A. Crooks

**Affiliations:** a Dept. of Pharm. Sciences, College of Pharmacy, University of Arkansas for Medical Sciences, Little Rock, AR 72205, USA; b Dept. of Chemistry, University of Kentucky, Lexington KY 40506, USA

**Keywords:** crystal structure, parthenolide, 2-iodo­aniline, Heck product

## Abstract

The title compound, C_21_H_25_NO_3_ [systematic name: (1a*R*,4*E*,7a*S*,8*E*,10a*S*,10b*R*)-8-(2-amino­benzyl­idene)-1a,5-dimethyl-2,3,6,7,7a,8,10a,10b-octa­hydro­oxireno[2′,3′:9,10]cyclo­deca­[1,2-*b*]furan-9(1a*H*)-one], was synthesized by the reaction of parthenolide [systematic name (1a*R*,7a*S*,10a*S*,10b*S*,*E*)-1a,5-dimethyl-8-methyl­ene-2,3,6,7,7a,8,10*a*,10b-octa­hydro­oxireno[2′,3′:9,10]cyclo­deca­[1,2-*b*]furan-9(1a*H*)-one] with 2-iodo­aniline *via* Heck reaction conditions. The mol­ecule is composed of fused ten-, five- (lactone), and three-membered (epoxide) rings. The lactone ring shows a flattened envelope-type conformation (r.m.s. deviation from planarity = 0.0477 Å), and bears a 2-amino­benzyl­idene substituent that is disordered over two conformations [occupancy factors 0.901 (4) and 0.099 (4)]. The ten-membered ring has an approximate chair–chair conformation. The dihedral angle between the 2-amino­benzyl­idine moiety (major component) and the lactone ring (mean plane) is 59.93 (7)°. There are no conventional hydrogen bonds, but there are a number of weaker C—H⋯O-type inter­actions.

## Abstract 2

The title mol­ecule is composed of fused ten-, five- (lactone), and three-membered (epoxide) rings. The lactone ring shows a flattened envelope-type conformation and bears a 2-amino­benzyl­idene substituent that is disordered over two conformations. The ten-membered ring has an approximate chair–chair conformation. There are no conventional hydrogen bonds, but there are a number of weaker C—H⋯O-type inter­actions.

## Chemical context   

Sesquiterpene lactones (SLs) are a large family of natural products that have been widely investigated for their anti­cancer activity. Parthenolide (PTL), a naturally occurring germacranolide SL (Minnaard *et al.*, 1999 ▸) isolated from the feverfew plant (*Tanacetum parthenium*) (Knight, 1995 ▸), has unique biological properties and selectively targets leukemia stem cells (LSC) compared to normal hematopoietic stem cells (Guzman *et al.*, 2005 ▸). PTL has been demonstrated to inhibit the NFkB pathway in LSCs, and also increases reactive oxygen species, and inhibits STAT3 (signal transduction and activation of transcription) (Mathema *et al.*, 2012 ▸). Synthetic analogues of SLs are also excellent sources of novel chemical entities for drug discovery, and over the last decade have been developed as efficacious anti­cancer drugs (Ghantous *et al.*, 2010 ▸). Previous work from our laboratory (Nasim & Crooks, 2008 ▸) reported the amino analogues of PTL as anti-leukemic agents, and moreover a water-soluble analogue of PTL, di­methyl­amino­parthenolide (DMAPT), has advanced into clinical studies (Ghantous *et al.*, 2010 ▸). Recently, Kempema *et al.* (2015 ▸) have reported C1 to C10-modified PTL analogues as anti-leukemic agents. Han *et al.* (2009 ▸) have also reported Heck products of PTL as anti-cancer agents. In continuing efforts from our group, Penthala *et al.* (2014*a* ▸) reported Heck products of PTL and Melampomagnolide B as anti-cancer agents. Subsequently, Bommagani *et al.* (2015 ▸) reported the crystal structure of (*E*)-13-(pyrimidin-5-yl)-parthenolide, an analog of PTL, which was found to have an *E*-configuration at C-13. The useful biological properties of PTL and its analogs directed our attention to design and synthesize novel bioactive analogs. In order to obtain detailed information on the structural conformation of the current mol­ecule and to determine the geometry of the exocyclic double bond, a single-crystal X-ray structure determination has been carried out.
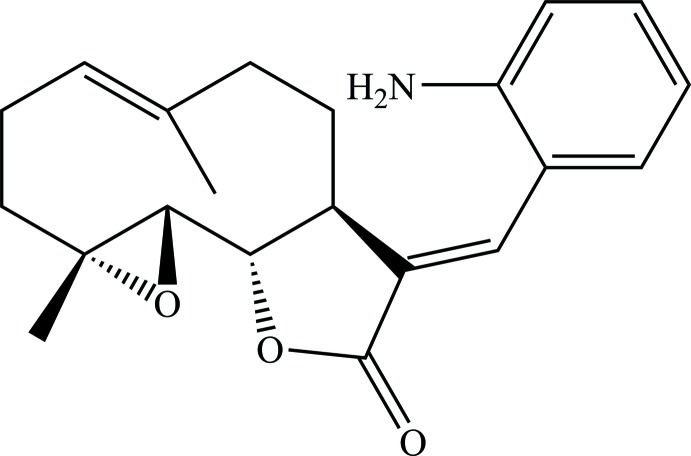


## Structural commentary   

**Figure 1:**
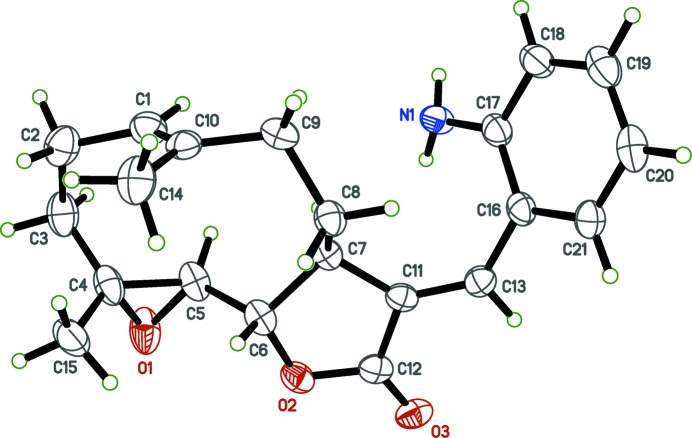
The mol­ecular structure of the title compound with ellipsoids drawn at the 50% probability level.

The title compound (Fig. 1 ▸) is built from the PTL substructure, which contains a ten-membered carbocyclic ring (chair–chair conformation) merged to a lactone ring, and an epoxide ring, as previously reported (Castañeda-Acosta *et al.*, 1993 ▸). The lactone ring has a flattened envelope-type conformation, wherein atoms C6 and C7 reside 0.093 (4) and −0.105 (4) Å above and below the mean plane through atoms C11, C12, O2, and O3. The mol­ecule also contains a 2-amino­benzyl­idene group attached by an *E*-exocyclic C11=C13 olefinic bond. The 2-amino­benzyl­idene ring is twisted out of the plane of the furan ring, subtending a dihedral angle of 59.93 (7)°. All other bond lengths and angles are largely unremarkable.

## Supra­molecular features   

**Figure 2:**
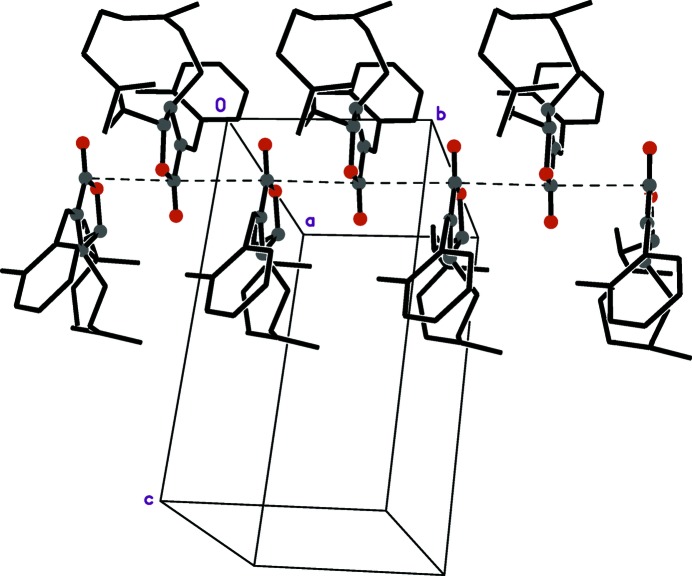
A packing plot showing the stacking of 2_1_ screw-related adjacent lactone groups. The stacking direction, shown by a dashed line, is parallel to the crystallographic *b* axis. For emphasis, lactone-group atoms are depicted as solid balls. For clarity, hydrogen atoms and minor disorder components are omitted.

There are no conventional hydrogen bonds in the crystal structure, although there are a number of weaker C—H⋯O-type inter­actions (Table 1 ▸). The most striking packing feature consists of 2_1_ screw-related (1 − *x*, 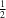 + *y*, −*z*) stacking of lactone groups parallel to the *b* axis (Fig. 2 ▸). The distance between planes of adjacent lactone rings is therefore half the *b*-axis length.

## Database survey   

A search of the November 2017 release (with three incremental updates) of the Cambridge Structure Database (Groom *et al.*, 2016 ▸) for the PTL substructure gave 30 hits. Three of these, PARTEN (Quick & Rogers, 1976 ▸), PARTEN01 (Bartsch *et al.*, 1983 ▸), and PARTEN02 (Long *et al.*, 2013 ▸) give the structure of PTL itself. One (EBOLOZ, Jamal *et al.*, 2014 ▸) is flagged in the CSD as a stereoisomer of parthenolide, though from the context it appears to be parthenolide with an incorrectly assigned absolute configuration. The remaining 26 are substituted variants of PTL. Of these, only six entries: HORZOF (Penthala *et al.*, 2014*b* ▸), HUKLAB, HUKLEF (Han *et al.*, 2009 ▸), QILGEZ (Penthala *et al.*, 2013 ▸), RUTPON (Bommagani *et al.*, 2015 ▸), and BEMHIN (El Bouakher *et al.*, 2017 ▸) are substituted at the exocyclic double bond.

## Synthesis and crystallization   

**Synthetic procedures:** The title compound, containing the PTL substructure, was synthesized by the previously reported literature procedure (Han *et al.*, 2009 ▸). In brief, parthenolide (1 mmol), 2-iodo­aniline (1.2 mmol), tri­ethyl­amine (3.0 mmol) and 5 mol% of palladium acetate were charged into di­methyl­formamide (2 ml) at room temperature. The reactants were stirred at 333–343 K for 24 h. After completion of the reaction, water was added to the reaction mass at room temperature, and the mixture was extracted into diethyl ether (2 × 30 mL). The combined organic layers were dried over anhydrous sodium sulfate, concentrated and purified by silica gel column chromatography.

**Crystallization:** The title compound was recrystallized from a mixture of hexane and acetone (9:1), which gave colorless crystals upon slow evaporation of the solution at room temperature over 24 h. Melting point 457–459 K. ^1^H NMR (400 MHz, CDCl_3_d): δ 7.63 (*s*, 1H), 7.17 (*d*, *J* = 6.4 Hz, 2H), 6.78 (*dd*, *J* = 7.6 Hz, *J* = 18.4 Hz, 2H), 5.26 (*d*, *J* = 11.6 Hz, 1H), 3.97 (*s*, 2H), 3.92 (*t*, *J* = 7.6 Hz, *J* = 15.6 Hz, 1H), 2.87–2.83 (*m*, 2H), 2.42–2.38 (*m*, 1H), 2.20–2.08 (*m*, 4H), 2.08–1.96 (*m*, 1H), 1.74 (*d*, *J* = 18.0 Hz, 1H), 1.63 (*s*, 3H), 1.36–1.26 (*m*, 4H) ppm; ^13^C NMR (100 MHz, CDCl_3_d) δ 171.17, 145.47, 135.37, 133.98, 131.11, 130.06, 129.05, 124.82, 118.93, 118.25, 116.21, 83.33, 66.91, 61.85, 47.42, 41.54, 36.49, 29.76, 24.42, 17.62, 17.54 ppm; (ESI): *m*/*z* C_21_H_26_NO_3_ [*M* + H] 340.28.

## Refinement details   

Crystal data, data collection and structure refinement details are summarized in Table 2 ▸. H atoms were found in difference-Fourier maps. Carbon-bound hydrogens were subsequently placed at idealized positions with constrained distances of 0.98 Å (*R*CH_3_), 0.99 Å (*R*_2_CH_2_), 1.00 Å (*R*_3_CH) and 0.95 Å (C*sp* ^2^H). Nitro­gen-bound hydrogens on the major disorder component were refined freely, while those on the minor component were heavily restrained. *U*_iso_(H) values were set to either 1.2*U*_eq_ or 1.5*U*_eq_ (*R*CH_3_) of the attached atom.

To ensure satisfactory refinement of disordered groups in the structure, a combination of constraints and restraints were employed. The constraints (*SHELXL* commands EXYZ and EADP) were used to fix parameters of superimposed or partially overlapping fragments. Restraints (*SHELXL* command SADI) were used to maintain the integrity of ill-defined or disordered groups. Refinement progress was checked using *PLATON* (Spek, 2009 ▸) and by an *R*-tensor (Parkin, 2000 ▸).

The minor component of disorder of the amine was apparent in a difference map. Given the small occupancy factor (only about 10%), the geometry of the minor component is approximate, and its hydrogen atoms were included merely to achieve the correct atom count.

The conventionally calculated Flack parameter does not convincingly indicate the proper assignment of absolute configuration. An alternative formulation of the chirality parameter using Parsons quotients (Parsons *et al.*, 2013 ▸) [the so-called ’*z*′ parameter = 0.07 (7)] as calculated by *PLATON* (Spek, 2009 ▸) is much more definitive.

## Supplementary Material

Crystal structure: contains datablock(s) I, global. DOI: 10.1107/S2056989018013622/sj5564sup1.cif

Structure factors: contains datablock(s) I. DOI: 10.1107/S2056989018013622/sj5564Isup2.hkl

CCDC reference: 1869537

Additional supporting information:  crystallographic information; 3D view; checkCIF report

## Figures and Tables

**Table 1:** Hydrogen-bond geometry (Å, °)

*D*—H⋯*A*	*D*—H	H⋯*A*	*D*⋯*A*	*D*—H⋯*A*
C5—H5⋯O3^i^	1.00	2.59	3.268 (3)	125
C7—H7⋯O3^i^	1.00	2.57	3.226 (3)	123
C15—H15*A*⋯O1^ii^	0.98	2.40	3.223 (3)	141

Symmetry codes: (i) 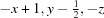; (ii) 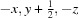.

**Table 2:** Experimental details

Crystal data	
Chemical formula	C_21_H_25_NO_3_
*M*_r_	339.42
Crystal system, space group	Monoclinic, *P*2_1_
Temperature (K)	90
*a*, *b*, *c* (Å)	11.6136 (3), 6.2403 (1), 12.6875 (3)
β (°)	104.385 (1)
*V* (Å^3^)	890.67 (3)
*Z*	2
Radiation type	Cu *K*α
μ (mm^−1^)	0.67
Crystal size (mm)	0.16 × 0.12 × 0.08

Data collection	
Diffractometer	Bruker X8 Proteum
Absorption correction	Multi-scan (*SADABS*; Krause *et al.*, 2015 ▸)
*T*_min_, *T*_max_	0.840, 0.942
No. of measured, independent and observed [*I* > 2σ(*I*)] reflections	24004, 2592, 2559
*R*_int_	0.041
(sin θ/λ)_max_ (Å^−1^)	0.602

Refinement	
*R*[*F* ^2^ > 2σ(*F* ^2^)], *wR*(*F* ^2^), *S*	0.032, 0.084, 1.08
No. of reflections	2592
No. of parameters	238
No. of restraints	2
H-atom treatment	H atoms treated by a mixture of independent and constrained refinement
Δρ_max_, Δρ_min_ (e Å^−3^)	0.18, −0.14
Absolute structure	Flack *x* determined using 811 quotients [(*I* ^+^)−(*I* ^−^)]/[(*I* ^+^)+(*I* ^−^)] (Parsons *et al.*, 2013 ▸), as calculated by *PLATON* (Spek, 2009 ▸).
Absolute structure parameter	0.07 (7)

Computer programs: *APEX2* and *SAINT* (Bruker, 2006 ▸), *XP* in *SHELXTL* and *SHELXS97* (Sheldrick, 2008 ▸), *SHELXL2013* (Sheldrick, 2015 ▸) and *CIFFIX* (Parkin, 2013 ▸).

## supplementary crystallographic information

### Crystal data

**Table:**

C_21_H_25_NO_3_	*F*(000) = 364
*M**_r_* = 339.42	*D*_x_ = 1.266 Mg m^−^^3^
Monoclinic, *P*2_1_	Cu *K*α radiation, λ = 1.54178 Å
*a* = 11.6136 (3) Å	Cell parameters from 9958 reflections
*b* = 6.2403 (1) Å	θ = 3.9–68.2°
*c* = 12.6875 (3) Å	µ = 0.67 mm^−^^1^
β = 104.385 (1)°	*T* = 90 K
*V* = 890.67 (3) Å^3^	Block, colourless
*Z* = 2	0.16 × 0.12 × 0.08 mm

### Data collection

**Table:**

Bruker X8 Proteum diffractometer	2592 independent reflections
Radiation source: fine-focus rotating anode	2559 reflections with *I* > 2σ(*I*)
Detector resolution: 5.6 pixels mm^-1^	*R*_int_ = 0.041
φ and ω scans	θ_max_ = 68.2°, θ_min_ = 3.6°
Absorption correction: multi-scan (*SADABS*; Krause *et al.*, 2015)	*h* = −13→13
*T*_min_ = 0.840, *T*_max_ = 0.942	*k* = −6→7
24004 measured reflections	*l* = −15→15

### Refinement

**Table:**

Refinement on *F*^2^	Secondary atom site location: difference Fourier map
Least-squares matrix: full	Hydrogen site location: mixed
*R*[*F*^2^ > 2σ(*F*^2^)] = 0.032	H atoms treated by a mixture of independent and constrained refinement
*wR*(*F*^2^) = 0.084	*w* = 1/[σ^2^(*F*_o_^2^) + (0.0449*P*)^2^ + 0.1894*P*] where *P* = (*F*_o_^2^ + 2*F*_c_^2^)/3
*S* = 1.08	(Δ/σ)_max_ < 0.001
2592 reflections	Δρ_max_ = 0.18 e Å^−^^3^
238 parameters	Δρ_min_ = −0.14 e Å^−^^3^
2 restraints	Absolute structure: Flack *x* determined using 811 quotients [(*I*^+^)-(*I*^-^)]/[(*I*^+^)+(*I*^-^)] (Parsons *et al.*, 2013), as calculated by PLATON (Spek, 2009).
Primary atom site location: structure-invariant direct methods	Absolute structure parameter: 0.07 (7)

### Special details

**Table:**

Experimental. The crystal was mounted with polyisobutene oil on the tip of a fine glass fibre, fastened in a copper mounting pin with electrical solder. It was placed directly into the cold stream of a liquid nitrogen based cryostat, according to published methods (Hope, 1994; Parkin & Hope, 1998). Diffraction data were collected with the crystal at 90K, which is standard practice in this laboratory for the majority of flash-cooled crystals.
Geometry. All esds (except the esd in the dihedral angle between two l.s. planes) are estimated using the full covariance matrix. The cell esds are taken into account individually in the estimation of esds in distances, angles and torsion angles; correlations between esds in cell parameters are only used when they are defined by crystal symmetry. An approximate (isotropic) treatment of cell esds is used for estimating esds involving l.s. planes.
Refinement. Refinement progress was checked using *Platon* (Spek, 2009) and by an *R*-tensor (Parkin, 2000). The final model was further checked with the IUCr utility *checkCIF*.

### Fractional atomic coordinates and isotropic or equivalent isotropic displacement parameters (Å^2^)

**Table:**

	*x*	*y*	*z*	*U*_iso_*/*U*_eq_	Occ. (<1)
O1	0.15259 (14)	0.2877 (3)	−0.01598 (12)	0.0449 (5)	
O2	0.37973 (12)	0.4845 (3)	0.01116 (10)	0.0310 (3)	
O3	0.57392 (13)	0.4878 (3)	0.08619 (10)	0.0338 (3)	
C1	0.0887 (2)	0.3088 (4)	−0.34185 (17)	0.0352 (5)	
H1	0.1376	0.1884	−0.3456	0.042*	
C2	−0.0258 (2)	0.2614 (4)	−0.31056 (19)	0.0398 (6)	
H2A	−0.0672	0.1415	−0.3555	0.048*	
H2B	−0.0781	0.3887	−0.3256	0.048*	
C3	−0.0032 (2)	0.2016 (5)	−0.18904 (19)	0.0408 (6)	
H3A	−0.0797	0.1974	−0.1680	0.049*	
H3B	0.0331	0.0573	−0.1769	0.049*	
C4	0.07850 (19)	0.3630 (4)	−0.11934 (17)	0.0353 (5)	
C5	0.20641 (18)	0.3246 (4)	−0.10509 (17)	0.0328 (5)	
H5	0.2251	0.1905	−0.1407	0.039*	
C6	0.29816 (17)	0.4982 (4)	−0.09641 (14)	0.0288 (4)	
H6	0.2587	0.6418	−0.1064	0.035*	
C7	0.37413 (16)	0.4677 (4)	−0.18048 (14)	0.0262 (4)	
H7	0.3626	0.3171	−0.2080	0.031*	
C8	0.34284 (18)	0.6194 (4)	−0.28030 (15)	0.0287 (5)	
H8A	0.3081	0.7530	−0.2596	0.034*	
H8B	0.4167	0.6578	−0.3014	0.034*	
C9	0.25450 (18)	0.5184 (4)	−0.37888 (14)	0.0314 (5)	
H9A	0.2521	0.6076	−0.4439	0.038*	
H9B	0.2840	0.3748	−0.3924	0.038*	
C10	0.12982 (17)	0.4963 (4)	−0.36473 (13)	0.0286 (4)	
C11	0.49909 (17)	0.4865 (4)	−0.11042 (13)	0.0255 (4)	
C12	0.49328 (19)	0.4875 (4)	0.00462 (15)	0.0280 (4)	
C13	0.60415 (17)	0.5038 (4)	−0.13427 (14)	0.0282 (4)	
H13	0.6711	0.5207	−0.0742	0.034*	
C14	0.0631 (2)	0.7032 (4)	−0.37465 (19)	0.0395 (6)	
H14A	−0.0194	0.6752	−0.3727	0.059*	
H14B	0.0644	0.7725	−0.4436	0.059*	
H14C	0.1005	0.7976	−0.3141	0.059*	
C15	0.0281 (2)	0.5807 (5)	−0.1117 (2)	0.0436 (6)	
H15A	−0.0376	0.5705	−0.0762	0.065*	
H15B	−0.0013	0.6402	−0.1850	0.065*	
H15C	0.0901	0.6745	−0.0690	0.065*	
C16	0.62889 (16)	0.5001 (4)	−0.24275 (14)	0.0279 (4)	
C17	0.58739 (18)	0.3343 (4)	−0.31796 (15)	0.0301 (4)	0.901 (4)
N1	0.52374 (18)	0.1616 (4)	−0.29365 (15)	0.0308 (5)	0.901 (4)
H1N	0.524 (3)	0.047 (5)	−0.334 (2)	0.046*	0.901 (4)
H2N	0.530 (3)	0.136 (6)	−0.225 (2)	0.046*	0.901 (4)
C17'	0.58739 (18)	0.3343 (4)	−0.31796 (15)	0.0301 (4)	0.099 (4)
H17'	0.5450	0.2160	−0.2992	0.036*	0.099 (4)
C18	0.61026 (19)	0.3482 (5)	−0.42116 (16)	0.0349 (5)	
H18	0.5830	0.2377	−0.4728	0.042*	
C19	0.67180 (19)	0.5197 (5)	−0.44896 (16)	0.0382 (6)	
H19	0.6843	0.5279	−0.5201	0.046*	
C20	0.71561 (19)	0.6804 (4)	−0.37407 (18)	0.0372 (5)	
H20	0.7588	0.7973	−0.3932	0.045*	
C21	0.69556 (18)	0.6680 (4)	−0.27111 (17)	0.0315 (5)	0.901 (4)
H21	0.7275	0.7751	−0.2188	0.038*	0.901 (4)
C21'	0.69556 (18)	0.6680 (4)	−0.27111 (17)	0.0315 (5)	0.099 (4)
N1'	0.7329 (16)	0.843 (3)	−0.2219 (13)	0.0308 (5)	0.099 (4)
H1N'	0.7209	0.8389	−0.1538	0.046*	0.099 (4)
H2N'	0.8119	0.8571	−0.2175	0.046*	0.099 (4)

### Atomic displacement parameters (Å^2^)

**Table:**

	*U*^11^	*U*^22^	*U*^33^	*U*^12^	*U*^13^	*U*^23^
O1	0.0318 (8)	0.0637 (13)	0.0419 (8)	0.0018 (9)	0.0144 (6)	0.0236 (8)
O2	0.0381 (7)	0.0293 (8)	0.0295 (6)	0.0003 (8)	0.0160 (5)	0.0015 (7)
O3	0.0452 (8)	0.0288 (8)	0.0258 (6)	−0.0091 (8)	0.0060 (6)	−0.0006 (7)
C1	0.0382 (12)	0.0304 (13)	0.0368 (10)	0.0021 (11)	0.0093 (9)	−0.0045 (9)
C2	0.0356 (12)	0.0331 (14)	0.0491 (12)	−0.0049 (10)	0.0075 (9)	0.0010 (10)
C3	0.0324 (11)	0.0388 (15)	0.0542 (13)	0.0006 (11)	0.0162 (9)	0.0129 (11)
C4	0.0310 (11)	0.0456 (15)	0.0331 (10)	0.0096 (11)	0.0154 (8)	0.0133 (10)
C5	0.0325 (10)	0.0298 (12)	0.0401 (10)	0.0067 (10)	0.0166 (8)	0.0112 (10)
C6	0.0311 (10)	0.0266 (11)	0.0314 (9)	0.0056 (10)	0.0130 (7)	0.0072 (9)
C7	0.0269 (9)	0.0251 (11)	0.0279 (8)	0.0011 (9)	0.0096 (7)	0.0014 (8)
C8	0.0277 (10)	0.0315 (12)	0.0282 (9)	0.0012 (9)	0.0094 (7)	0.0052 (8)
C9	0.0381 (11)	0.0335 (13)	0.0246 (8)	0.0022 (10)	0.0114 (7)	−0.0011 (9)
C10	0.0314 (10)	0.0319 (12)	0.0205 (8)	0.0005 (11)	0.0029 (7)	−0.0023 (9)
C11	0.0320 (10)	0.0204 (10)	0.0251 (8)	0.0004 (9)	0.0090 (7)	−0.0003 (8)
C12	0.0389 (10)	0.0178 (10)	0.0287 (8)	−0.0027 (10)	0.0110 (7)	0.0005 (9)
C13	0.0277 (9)	0.0281 (11)	0.0276 (8)	0.0002 (10)	0.0048 (7)	−0.0002 (9)
C14	0.0334 (11)	0.0383 (15)	0.0446 (12)	0.0063 (11)	0.0058 (9)	0.0133 (11)
C15	0.0393 (12)	0.0528 (17)	0.0417 (12)	0.0142 (12)	0.0161 (9)	−0.0052 (11)
C16	0.0221 (8)	0.0335 (12)	0.0285 (8)	0.0047 (10)	0.0071 (7)	0.0024 (9)
C17	0.0266 (9)	0.0338 (12)	0.0314 (9)	0.0032 (10)	0.0103 (7)	−0.0003 (9)
N1	0.0382 (11)	0.0292 (12)	0.0270 (9)	−0.0012 (9)	0.0122 (7)	−0.0032 (8)
C17'	0.0266 (9)	0.0338 (12)	0.0314 (9)	0.0032 (10)	0.0103 (7)	−0.0003 (9)
C18	0.0333 (10)	0.0435 (15)	0.0299 (9)	0.0041 (11)	0.0119 (8)	−0.0012 (10)
C19	0.0331 (10)	0.0509 (17)	0.0332 (10)	0.0071 (11)	0.0134 (8)	0.0107 (11)
C20	0.0274 (10)	0.0412 (15)	0.0456 (12)	0.0013 (11)	0.0140 (8)	0.0125 (11)
C21	0.0225 (9)	0.0351 (13)	0.0371 (10)	0.0021 (9)	0.0075 (7)	0.0011 (9)
C21'	0.0225 (9)	0.0351 (13)	0.0371 (10)	0.0021 (9)	0.0075 (7)	0.0011 (9)
N1'	0.0382 (11)	0.0292 (12)	0.0270 (9)	−0.0012 (9)	0.0122 (7)	−0.0032 (8)

### Geometric parameters (Å, º)

**Table:**

O1—C5	1.440 (2)	C9—H9B	0.9900
O1—C4	1.456 (2)	C10—C14	1.496 (3)
O2—C12	1.342 (3)	C11—C13	1.332 (3)
O2—C6	1.458 (2)	C11—C12	1.478 (2)
O3—C12	1.211 (3)	C13—C16	1.474 (2)
C1—C10	1.323 (4)	C13—H13	0.9500
C1—C2	1.508 (3)	C14—H14A	0.9800
C1—H1	0.9500	C14—H14B	0.9800
C2—C3	1.544 (3)	C14—H14C	0.9800
C2—H2A	0.9900	C15—H15A	0.9800
C2—H2B	0.9900	C15—H15B	0.9800
C3—C4	1.510 (4)	C15—H15C	0.9800
C3—H3A	0.9900	C16—C21	1.402 (3)
C3—H3B	0.9900	C16—C17	1.409 (3)
C4—C5	1.471 (3)	C17—N1	1.384 (3)
C4—C15	1.492 (4)	C17—C18	1.402 (3)
C5—C6	1.504 (3)	N1—H1N	0.88 (3)
C5—H5	1.0000	N1—H2N	0.87 (3)
C6—C7	1.556 (2)	C18—C19	1.381 (4)
C6—H6	1.0000	C18—H18	0.9500
C7—C11	1.506 (3)	C19—C20	1.388 (4)
C7—C8	1.550 (3)	C19—H19	0.9500
C7—H7	1.0000	C20—C21	1.385 (3)
C8—C9	1.541 (3)	C20—H20	0.9500
C8—H8A	0.9900	C21—H21	0.9500
C8—H8B	0.9900	N1'—H1N'	0.9100
C9—C10	1.509 (3)	N1'—H2N'	0.9100
C9—H9A	0.9900		
			
C5—O1—C4	61.05 (13)	C10—C9—H9B	108.8
C12—O2—C6	111.18 (13)	C8—C9—H9B	108.8
C10—C1—C2	128.4 (2)	H9A—C9—H9B	107.7
C10—C1—H1	115.8	C1—C10—C14	125.06 (19)
C2—C1—H1	115.8	C1—C10—C9	121.1 (2)
C1—C2—C3	111.59 (19)	C14—C10—C9	113.8 (2)
C1—C2—H2A	109.3	C13—C11—C12	119.54 (17)
C3—C2—H2A	109.3	C13—C11—C7	132.42 (16)
C1—C2—H2B	109.3	C12—C11—C7	108.03 (16)
C3—C2—H2B	109.3	O3—C12—O2	120.69 (17)
H2A—C2—H2B	108.0	O3—C12—C11	128.95 (19)
C4—C3—C2	110.5 (2)	O2—C12—C11	110.35 (16)
C4—C3—H3A	109.6	C11—C13—C16	127.78 (17)
C2—C3—H3A	109.6	C11—C13—H13	116.1
C4—C3—H3B	109.6	C16—C13—H13	116.1
C2—C3—H3B	109.6	C10—C14—H14A	109.5
H3A—C3—H3B	108.1	C10—C14—H14B	109.5
O1—C4—C5	58.93 (13)	H14A—C14—H14B	109.5
O1—C4—C15	112.9 (2)	C10—C14—H14C	109.5
C5—C4—C15	122.7 (2)	H14A—C14—H14C	109.5
O1—C4—C3	117.2 (2)	H14B—C14—H14C	109.5
C5—C4—C3	115.6 (2)	C4—C15—H15A	109.5
C15—C4—C3	116.50 (19)	C4—C15—H15B	109.5
O1—C5—C4	60.02 (12)	H15A—C15—H15B	109.5
O1—C5—C6	119.7 (2)	C4—C15—H15C	109.5
C4—C5—C6	124.6 (2)	H15A—C15—H15C	109.5
O1—C5—H5	114.0	H15B—C15—H15C	109.5
C4—C5—H5	114.0	C21—C16—C17	119.53 (17)
C6—C5—H5	114.0	C21—C16—C13	118.4 (2)
O2—C6—C5	108.06 (16)	C17—C16—C13	122.1 (2)
O2—C6—C7	106.71 (15)	N1—C17—C18	119.5 (2)
C5—C6—C7	111.96 (18)	N1—C17—C16	122.18 (18)
O2—C6—H6	110.0	C18—C17—C16	118.4 (2)
C5—C6—H6	110.0	C17—N1—H1N	115 (2)
C7—C6—H6	110.0	C17—N1—H2N	117 (2)
C11—C7—C8	115.51 (17)	H1N—N1—H2N	115 (3)
C11—C7—C6	102.31 (14)	C19—C18—C17	121.1 (2)
C8—C7—C6	115.32 (17)	C19—C18—H18	119.4
C11—C7—H7	107.7	C17—C18—H18	119.4
C8—C7—H7	107.7	C18—C19—C20	120.67 (19)
C6—C7—H7	107.7	C18—C19—H19	119.7
C9—C8—C7	112.77 (18)	C20—C19—H19	119.7
C9—C8—H8A	109.0	C21—C20—C19	119.1 (2)
C7—C8—H8A	109.0	C21—C20—H20	120.4
C9—C8—H8B	109.0	C19—C20—H20	120.4
C7—C8—H8B	109.0	C20—C21—C16	121.1 (2)
H8A—C8—H8B	107.8	C20—C21—H21	119.4
C10—C9—C8	113.94 (16)	C16—C21—H21	119.4
C10—C9—H9A	108.8	H1N'—N1'—H2N'	109.5
C8—C9—H9A	108.8		
			
C10—C1—C2—C3	−107.8 (3)	C8—C9—C10—C1	−103.1 (2)
C1—C2—C3—C4	49.0 (3)	C8—C9—C10—C14	74.6 (2)
C5—O1—C4—C15	−115.5 (2)	C8—C7—C11—C13	−43.4 (4)
C5—O1—C4—C3	104.9 (2)	C6—C7—C11—C13	−169.5 (3)
C2—C3—C4—O1	−151.63 (19)	C8—C7—C11—C12	135.47 (19)
C2—C3—C4—C5	−85.0 (2)	C6—C7—C11—C12	9.4 (2)
C2—C3—C4—C15	70.2 (2)	C6—O2—C12—O3	176.5 (2)
C4—O1—C5—C6	115.1 (3)	C6—O2—C12—C11	−4.5 (3)
C15—C4—C5—O1	98.8 (2)	C13—C11—C12—O3	−5.6 (4)
C3—C4—C5—O1	−107.7 (2)	C7—C11—C12—O3	175.3 (2)
O1—C4—C5—C6	−107.3 (2)	C13—C11—C12—O2	175.4 (2)
C15—C4—C5—C6	−8.5 (3)	C7—C11—C12—O2	−3.6 (3)
C3—C4—C5—C6	145.1 (2)	C12—C11—C13—C16	178.2 (2)
C12—O2—C6—C5	131.12 (19)	C7—C11—C13—C16	−3.0 (4)
C12—O2—C6—C7	10.6 (3)	C11—C13—C16—C21	127.5 (3)
O1—C5—C6—O2	44.4 (3)	C11—C13—C16—C17	−52.3 (3)
C4—C5—C6—O2	116.6 (2)	C21—C16—C17—N1	178.3 (2)
O1—C5—C6—C7	161.60 (18)	C13—C16—C17—N1	−1.9 (3)
C4—C5—C6—C7	−126.2 (2)	C21—C16—C17—C18	−2.7 (3)
O2—C6—C7—C11	−11.8 (2)	C13—C16—C17—C18	177.11 (19)
C5—C6—C7—C11	−129.85 (19)	N1—C17—C18—C19	179.0 (2)
O2—C6—C7—C8	−138.03 (19)	C16—C17—C18—C19	−0.1 (3)
C5—C6—C7—C8	103.9 (2)	C17—C18—C19—C20	1.8 (3)
C11—C7—C8—C9	146.42 (18)	C18—C19—C20—C21	−0.8 (3)
C6—C7—C8—C9	−94.4 (2)	C19—C20—C21—C16	−2.0 (3)
C7—C8—C9—C10	71.1 (2)	C17—C16—C21—C20	3.8 (3)
C2—C1—C10—C14	−6.3 (4)	C13—C16—C21—C20	−176.0 (2)
C2—C1—C10—C9	171.1 (2)		

### Hydrogen-bond geometry (Å, º)

**Table:**

*D*—H···*A*	*D*—H	H···*A*	*D*···*A*	*D*—H···*A*
C5—H5···O3^i^	1.00	2.59	3.268 (3)	125
C7—H7···O3^i^	1.00	2.57	3.226 (3)	123
C15—H15*A*···O1^ii^	0.98	2.40	3.223 (3)	141

Symmetry codes: (i) −*x*+1, *y*−1/2, −*z*; (ii) −*x*, *y*+1/2, −*z*.
